# Survival of primary ankle replacements: data from global joint registries

**DOI:** 10.1186/s13047-022-00539-2

**Published:** 2022-05-07

**Authors:** Thomas A. Perry, Alan Silman, David Culliford, Lucy Gates, Nigel Arden, Catherine Bowen, the International Ankle Arthroplasty Registry Consortium, Ian A. Harris, Chelsea Nicole Dyer, Andrew Beischer, Ilana Ackerman, Ove Furnes, Geir Hallan, Keijo T. Mäkelä, Miika Stenholm, Anders Henricson, John McKie, Dawson Muir

**Affiliations:** 1 Nuffield Department of Orthopaedics, Rheumatology and Musculoskeletal Sciences, Botnar Research Centre, University of Oxford, Old Road, Oxford, OX3 7LD UK; 2 Centre for Sport, Exercise and Osteoarthritis Versus Arthritis, Nuffield Department of Orthopaedics, Rheumatology and Musculoskeletal Sciences, University of Oxford, Oxford, UK; 3 Kennedy Institute of Rheumatology, University of Oxford, Roosevelt Drive, Headington, Oxford, OX3 7FY UK; 4 NIHR Applied Research Collaboration (ARC) Wessex, School of Health Sciences, University of Southampton, Southampton, UK; 5 School of Health Sciences, Faculty of Environmental and Life Sciences, University of Southampton, Southampton, UK; 6 Centre for Sport, Exercise and Osteoarthritis Versus Arthritis, University of Southampton, Southampton, UK; 7 MRC Lifecourse Epidemiology Unit, Southampton General Hospital, University of Southampton, Southampton, UK

**Keywords:** Ankle, Revision, Registries, Annual, Incidence, Survival

## Abstract

**Background:**

Ankle arthroplasty, commonly known as ankle replacement, is a surgical procedure for treating end-stage ankle osteoarthritis. Whilst evidence shows good clinical results after surgery, little is known of the long-term survival of ankle replacements and the need for ankle revision. Using more recent implant data and long-term data, there is now opportunity to examine at a population-level the survival rate for ankle implants, to examine between-country differences in ankle revision surgery, and to compare temporal trends in revision rates between countries.

**Methods:**

Four national joint registries from Australia, New Zealand, Norway and Sweden provided the necessary data on revision outcome following primary ankle replacement, for various periods of observation – the earliest starting in 1993 up to the end of 2019. Data were either acquired from published, online annual reports or were provided from direct contact with the joint registries. The key information extracted were Kaplan-Meier estimates to plot survival probability curves following primary ankle replacement.

**Results:**

The survival rates varied between countries. At 2 years, across all registries, survival rates all exceeded 0.9 (range 0.91 to 0.97). The variation widened at 5 years (range 0.80 to 0.91), at 10 years (range 0.66 to 0.84) and further at 15-years follow-up (0.56 to 0.78). At each time point, implant survival was greater in Australia and New Zealand with lower rates in Sweden and Norway.

**Conclusions:**

We observed variation in primary ankle replacement survival rates across these national registries, although even after 5 years, these population derived data show an 80% revision free survival. These data raise a number of hypotheses concerning the reasons for between-country differences in revision-free survival which will require access to primary data for analysis.

**Supplementary Information:**

The online version contains supplementary material available at 10.1186/s13047-022-00539-2.

## Introduction

In the last decade total ankle replacement (TAR) has gained popularity as a surgical method of treating end-stage ankle osteoarthritis (OA) over ankle arthrodesis (fusion) [1–3]. Comparative studies of arthrodesis and ankle replacement have, however, revealed that rates of re-operation and major complications are consistently higher after ankle replacement [4–8]. Some of the major complications associated with failure of the joint replacement include infections and loosening of the components, and indications for ankle revision have been shown to vary considerably between countries [9]. Bartel and Roukis described in 2013, using data from six national ankle joint registries, that survival rates for TAR were high in the short-term (i.e. 2–10 years) with revision free survival exceeding 80% at 10 years follow-up [10]. In the same study [10] and using data from a separate study of ankle replacement survival [9], primary ankle replacement survival has been shown to vary considerably between countries with 5-year survival rates as low as 78–81% in Sweden and as high as 93% in New Zealand [9, 10]. There is, however, a need for the assessment of ankle replacement survival in the long term (i.e. > 10 years) and to make comparisons between countries. A single study using data from the Swedish Ankle registry showed that the survival rate of ankle replacements at 15 years was 0.63 (95% confidence interval (CI) 0.58 to 0.67) and at 20 years was 0.58 (95% CI 0.52 to 0.65) [11]. Compared to hip and knee replacements, rates of survival for primary ankle replacements are relatively low especially in the long-term [12, 13].

Currently, six countries have joint specific and/or national joint registries which capture data relating to the ankle joint. Previous studies, using data from national joint registries (NJRs), have shown that revision of a primary ankle replacement is not uncommon. There is now opportunity to retrospectively examine the survival of primary ankle replacements and the need for revision in the long term as we now have more years of follow-up and more recent implant data. A better understanding of the differences between countries in survival of primary ankle replacement will help inform patient expectations. Our aims, therefore, were to examine the long-term survival of primary ankle replacements, to compare temporal trends in revision rates between countries, and to examine between-country differences in ankle revision surgery by assessing the cumulative rate of ankle revisions.

## Methods and materials

We identified six joint registries with ankle joint data using the membership list of the International Society of Arthroplasty Registries (ISAR) [14]. Finland, Sweden, Norway, New Zealand (NZ), Australia, and the United Kingdom (UK) collect data for the ankle joint from their respective national joint registries; i) Finnish Arthroplasty Register (FAR) [15] (inception year: 1980), ii) The Swedish Ankle Registry (SwedAnkle) [16] (inception year: 1993), iii) The Norwegian Arthroplasty Register [17] (inception year: 1994), iv) New-Zealand Orthopaedic Association Joint Registry (NZOA Joint Registry) [18] (inception year: 2000), v) Australian Orthopaedic Association National Joint Replacement Registry (AOANJRR) [19] (inception year: 2007) and vi) National Joint Registry for England, Wales, Northern Ireland, the Isle of Man and the States of Guernsey (UK-NJR) [20, 21] (inception year: 2010).

In the current study, we used data from Australia, New Zealand, Norway and Sweden which have routinely available survival data; specifically, each national joint registry was evaluated for the availability of Kaplan-Meier (KM) estimates and other primary ankle replacement survival data (e.g. life tables). Finland was not included in the current study due to local concerns about the completeness of the data. Further, although the UK-NJR collected data on the outcome, it was unable to provide the survival analysis requested. Although there were slight variations between the four contributing registries in the definitions of ankle revision/survival of primary replacement [10, 22], most adhered to a single definition: “the retention of the primary ankle replacement until either the prosthesis (total or part) was revised, removed or exchanged”. Differences in the revision definitions were observed with Sweden defining a revision as the ‘exchange or extraction of 1 or more of the 3 prosthetic components with the exception of incidental exchange of the PE insert’ and Australia including ‘reoperations of primary partial, primary total, or previous revision procedures’ [10].

Life tables were generated from population-level summary statistics based on the number at risk at the beginning of each year of follow up following surgery, together with the number censored because of death or revision. KM estimates at follow up were extracted from published, electronically available annual reports or summary tables that were available either on the registry websites or were made available by direct contact with the respective registries (data available online was correct as of May 1st 2021). Data that were not open access was requested where possible from the host registry.

### Kaplan-Meier survival analysis

We sought to examine the survival of primary ankle replacements by recreating KM survival curves for all registries. Here, life tables and KM estimates were used to estimate the survival rates at a population level as individual patient-level data were not available for all registries. The survival rates were calculated and graphically displayed as KM curves which show the probability of an event (i.e. survival of primary ankle replacement) occurring over time. Although baseline data (e.g. 0-years follow-up) were not available for all registries, the survival rate was assumed to be equal to 1 at 0-years follow-up. After each time increment (1-year), patients who were lost to follow-up, withdrew before the event of interest (e.g. emigrated) or died were censored. Separate counts for censored events were not reported for all registries. If KM estimators were not already calculated and reported in the annual reports, they were calculated for each 1-year interval using the formula below;

$$ {\hat{S}}_{t_i+1}={\hat{S}}_{t_i}\times \left({n}_{t_i+1}-{d}_{t_i+1}\right)/{n}_{t_i+1} $$

$\hat{S}(t)$ = survival function, *t*_*i*_ = a time when at least one event happened, *d*_*i*_ = number of events (e.g., ankle revisions) that happened at time *t*_*i*_ and, *n*_*i*_ the individuals known to have survived (i.e. at risk and not yet had an event, died or been censored) up to time *t*_*i*_.Whilst KM estimates were available for up to 25-years follow-up in a single registry (Norway), we only used data where the numbers at risk for revision at the beginning of the given year were equal to or greater than 10 patients.

## Results

Data on primary replacement survival were available on just under 6700 patients for the period from 1993 to 2019. During this time, a total of 1080 ankle revisions were performed; see Table 1. Of the four registries, one had data for up to 13 years follow-up after primary ankle replacement (Australia) whilst three had survival data for ≥19 years follow-up (New Zealand, Norway and Sweden).

**Table 1:** Summary statistics for survival of primary ankle replacements across national joint registries

Country	Registry Inception	Survival: Years of follow-up available	Survival: Period Used (years)	Number of Primaries	Number of Revisions
Australia	2007^a^	13	12	2564	250
New Zealand	2000^b^	20	19	1737	191
Norway	1994^c^	25	20	1197	370
Sweden	1993^d^	20	19	1198	269

^a^January 2007 to 31st December 2019: data provided directly from AOANJRR

^b^January 2000 to December 2019: data provided directly from NZOA

^c^1994 to 2019: data provided directly from Norway

^d^1993 to 31st December 2019: data provided directly from SwedAnkle

Finland was not included given local concerns about the completeness of the data. Further, although the UK-NJR collected data on the outcome, it was unable to provide the survival analysis requested.

### Temporal trends in survival of primary ankle replacement

**Fig. 1:**
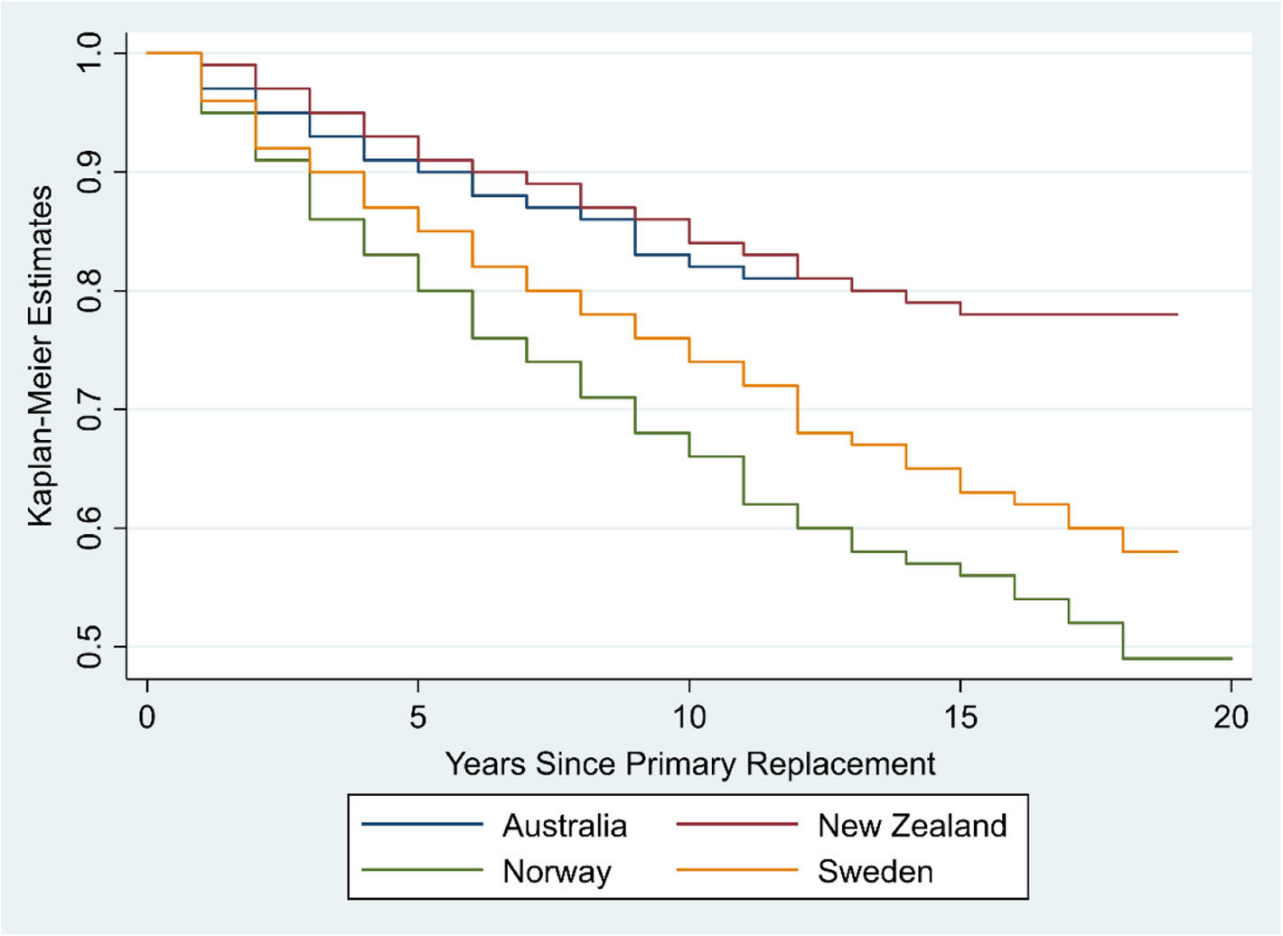
Plot of Kaplan-Meier estimates against years since primary ankle replacement across four joint registries

Of the four registries, all demonstrated a gradual decline in primary ankle replacement survival over the follow-up period. Primary ankle replacement survival rates were similar up to 2-years follow-up however, trends were highly divergent after this point. We plotted the calculated KM estimates against years since primary ankle replacement; see Fig. 1. Of the four registries assessed, all had different inception dates with the Scandinavian registries starting recruitment from 1993 to 1994, and Australia and New Zealand starting in 2007 and 2000 respectively. Therefore, survival years may not correspond to the same years of study across registries.

### Survival of primary ankle replacement

Using data from all registries, the mean (range) primary ankle replacement survival rate at 2-years was 0.94 (0.91 to 0.97), 0.86 (0.80 to 0.91) at 5 years, 0.82 (0.74 to 0.89) at 7-years and 0.77 (0.66 to 0.84) at 10-years follow-up. In the long-term and using data from 3 registries (New Zealand, Norway and Sweden), the mean primary ankle replacement survival rate was 0.66 (0.56 to 0.78) at 15-years and 0.62 (0.49 to 0.78) at 19-years follow-up. Australia and New Zealand demonstrated the highest rates of ankle replacement survival both in the short and long-term.

## Discussion

The purpose of the current study was to examine the long-term survival of ankle replacements, to examine between-country differences in ankle revision surgery, and to compare temporal trends in revision rates between countries. Across the four joint registries used, we observed between-country variation in survival rates in both the short-term (2–10 years) and in the long-term (> 10 years).

Few studies have examined and compared ankle replacement survival using data from national joint registries [9, 10]. We go beyond these studies to examine primary ankle replacement survival for a longer follow-up period (up to 20 years) and using the most complete and up to date data. This allowed us to examine temporal trends using robust country-level data. Our estimates of ankle replacement survival are similar to those previously reported by Bartel and Roukis [10]; 0.94 vs. 0.94 at 2 years, 0.87 vs. 0.86 at 5 years, and 0.81 vs. 0.77 at 10 years [10]. Using more current data, we were able to examine survival rates in the long-term at 15- and 19-years follow-up. Compared to the most recent data on primary ankle replacement survival in the long-term, our estimates of primary ankle replacement survival were comparable. For instance, a single study which examined long-term ankle replacement survival in Sweden, survival was reported as 0.63 (CI 0.58–0.67) at 15-years and 0.58 (CI 0.52–0.65) at 20 years [11].

We observed between-country differences in primary ankle replacement survival. The available data did not permit any adjustment for age and gender differences in the population receiving implants. Another possible explanation is that there are different thresholds (e.g. surgical requirements) for revision in different countries although there are, to our knowledge, no national guidelines governing indications for revision – future work in the field should include the development standardised indications for revision. Further, the failure mechanisms which lead to ankle revision are highly contested. There are several factors outside of the ankle replacement which are likely to influence rates of ankle revision and primary ankle replacement survival. For instance, improved patient selection (e.g. age at intervention) [23–25], type of implant used, frequency of primary replacement and, surgical caseload and skill [26, 27] affect revision and survival rates. This suggests that risk factors for ankle revision following primary ankle replacement may be, to some extent, modifiable: that is, if countries with high revision rates adopt the practices of countries with low revision rates, the full benefits of a primary ankle replacement may be gained without the consequence of high rates of revision/poor replacement survival. Specialist centres for the management of ankle replacements may facilitate improved survival rates.

Temporal changes in disease indications, such as a decline in severe destructive rheumatoid arthritis (RA) [28], as well as within country demographic shift for example operating on older people are also likely to influence implant survival rates.

We also considered differences in the indications for revision, which dependent on the registry, fell into one of the following six categories: 1) fracture / dislocation, 2) pain, 3) instability / reduced mobility, 4) prosthesis issues, 5) pathology and 6) ‘other’ (see Supplementary 1). We were unable to undertake a robust analysis of differences between registries in the proportions with these indications. We suggest that there is an international coordinated effort to harmonise the coding of these indications. A quick analysis suggested that ankle pain, prosthesis issues (e.g. loosening, defective polyethylene) and malalignment/fracture were among the most common reasons for revision which would need further exploration with higher quality data.

The use of different ankle prostheses will also give rise to different survival rates. For instance, in the current analysis implants from Australia and New Zealand demonstrated greater levels of survival compared to those from Norway and Sweden. These lower levels of survival in Scandinavia, to some extent, may be explained by the more long-term use of the early Scandinavian Total Ankle Replacement (STAR) design, with higher rates of prosthesis loosening reported for the first-generation LINK® STAR prothesis compared to the second-generation prosthesis [29].

There are other limitations to this study which require careful consideration. For instance, we assessed ankle replacement survival at a population-level as patient-level statistics were not available. Subsequently, we analysed the number of replacements rather than the number of patients; we were unable to report on whether one patient had multiple revisions. Whilst this method agrees with previous studies and has been shown to have little effect on the accuracy of survival estimates [11], we acknowledge that this may limit the generalizability of our findings. In addition, there is a degree of uncertainty regarding the reporting of the registry data. For instance, there may be under-reporting of either primary surgery and/or revision to these registers. It is unclear whether the counts of primaries and/or revisions reported here are matched within individuals (i.e. revisions may be reported in people who did not have their primary surgery entered and vice-versa). A high level of data completeness for the capture of primary ankle replacements has, however, been previously reported for the period investigated in the current study [30].

There are challenges to using ‘revision’ as an endpoint due to varying between-registry definitions. Whilst the included registries were similar in their definitions of the primary endpoint [10], slight variations were apparent between registries. For instance, the Norwegian registry counts all re-operations, including debridements, as revisions whilst in other registries ‘debridement’ is not specified as a revision. Such variations could give rise to differences in the estimates of annual incidence, particular if ‘revisions’ include re-operations; the challenges of registry terminology have been reported previously [22, 31]. More so, variations in the definitions of disease indications will also influence ankle replacement survival rates. Future work should aim to harmonise registry definitions of both replacement, revision and disease indications which could be achieved through consensus study of the international datasets.

We did not assess ankle replacement survival and rates of revision by model of implant used. One of the main surgical factors which has been associated with ankle replacement survival rates is the model/type of implant used. There is evidence, using data from joint registries, to suggest that more modern ankle replacements have better rates of survival at 5 (0.81 vs. 0.88) and 10-years (0.69 vs. 0.84) follow-up compared to older prosthesis designs [11]. The speed at which new implants are introduced and the time required for surgical training and education will influence the need for revision and subsequently, survival of primary ankle replacements. Specifically, any benefits of more modern implants may not be observed for several years after introduction, following a period of surgical learning and national adoption. There was evidence of this in the current study, specifically one of the oldest joint registries, Sweden, has only recently started to show a decline in the annual incidence of total ankle revisions. In the current study, we did not request data on the number of revisions by implant type due to the low counts of total replacements; we were concerned that small numbers and confounding by surgeon and year of operation would prevent meaningful analysis of replacement survival by implants. In addition, we were unable to examine generational differences in survival rates because we did not have data on the date of first acquisition of survival data. Subsequently, we were unable to examine secular trends between registries as we were unable to compare calendar years. Lastly, we did not undertake formal significant testing to compare the curves between the 4-time series, as the results from such an exercise are complex to interpret and would have added little to the interpretation of the results from simply comparing the shape of each country’s curve. Future work aims to compare secular trends across registries during which both time-dependant and implant-dependant factors are less likely to affect survival rates.

In addition to surgical factors, the between-country differences in rates of primary ankle replacement will vary by population size and demographic structure. There is variation in the age structure of these populations with New Zealand for example having the lowest proportion aged over 65 [32]. Thus, even without formal age adjustment the between-country differences do not appear to be explained by age. There are limitations to using KM estimates to examine joint replacement survival. For instance, it is assumed that the survival probabilities are the same for patients who entered the registry at study inception compared to patients who entered more recently [33]. This assumption may not hold true due to the continuing improvement and safety of ankle implants with the introduction of new replacements over time.

## Conclusion

Using data from national joint registries, there was variation between countries in short-term and long-term survival rates for primary ankle replacement. Differences in the dates of registry inception and capture rates of revision are likely reasons for such variation. Our findings also show a consistent trend towards a gradual decline in ankle replacement survival between 5 and 19 years after primary joint replacement.

## Supplementary Information

**Additional file 1: Supplementary 1.** Classification of disease indications for ankle revision.

## Data Availability

The datasets used in the current study are not publicly available due to data protection purposes but are available on reasonable request. Access to data generated in this report should be sent to the corresponding author at thomas.perry@kennedy.ox.ac.uk whilst requests for the individual registry data should be submitted to the principal investigators of the respective registries.

## Abbreviations

OA: osteoarthritis
RA: rheumatoid arthritis
NZ: New Zealand
UK: United Kingdom
KM: Kaplan-Meier
TAR: total ankle replacement
NJRs: national joint registries
ISAR: the International Society of Arthroplasty Registries
FAR: Finnish Arthroplasty Register
SwedAnkle: The Swedish Ankle Registry
NZOA Joint Registry: New-Zealand Orthopaedic Association Joint Registry
AOANJRR: Australian Orthopaedic Association National Joint Replacement Registry
UK-NJR: National Joint Registry for England, Wales, Northern Ireland, the Isle of Man and the States of Guernsey
