# Supplementary material for: Survival of primary ankle replacements: data from global joint registries
Source: J Foot Ankle Res. 2022 May 7;15:33. doi: 10.1186/s13047-022-00539-2 (PMC9078004; doi:10.1186/s13047-022-00539-2)
Supplement: Supplementary file 1 — Additional file 1: Supplementary 1. Classification of disease indications for ankle revision. [file 13047_2022_539_MOESM1_ESM.docx]

| **Supplementary 1:** Classification of disease indications for ankle revision. | | | | |  | | |  |
| --- | --- | --- | --- | --- | --- | --- | --- | --- |
| **Country** | **Fracture /**  **Dislocation** | **Pain** | **Instability /**  **reduced mobility** | **Prosthesis**  **Issues** | | **Pathology** | **Other** | |
| Australia | Fracture | Pain | Instability  Malalignment | Implant Breakage Ankle Insert  Incorrect Sizing  Loosening  Prosthesis Dislocation  Prosthesis Dissociation  Wear Ankle insert | | Arthrofibrosis  Infection  Lysis  Metal Related Pathology  Osteonecrosis  Synovitis | Heterotopic bone  Impingement  Tumour  Other | |
| New Zealand | Fracture – talus  Dislocation | Pain | - | Loosening - Talar Component  Loosening- Tibia | | Deep infection | Other | |
| Norway | Fracture (Near implant)  Dislocation | Pain | Instability  Poor mobility  Malalignment | Loose - proximal component  Loose - distal component  Defect Polyethylene | | Deep infection  Osteolysis | Other  Missing | |
| Sweden | Plastic wear / fracture  Fracture / dislocation | Intractable Pain | Instability  Varus  Valgus | Aseptic Loosening  Technical error | | Infection | Miscellaneous / other | |
